# Supplementary material for: Impact of body and orofacial appearance on life satisfaction among Brazilian adults
Source: PLoS One. 2022 Nov 4;17(11):e0275728. doi: 10.1371/journal.pone.0275728 (PMC9635715; doi:10.1371/journal.pone.0275728)
Supplement: S1 Table — (DOCX) [file pone.0275728.s001.docx]

**S1 Table. Psychometric properties of the instruments fitted to the study samples.**

| **Scale** | **Dimension(s)** | **Male Sample (n=580)** | | | | | **Female Sample (n=1,940)** | | | | |
| --- | --- | --- | --- | --- | --- | --- | --- | --- | --- | --- | --- |
|  |  | **CFI** | **TLI** | **RMSEA** | **SRMR** | **α** | **CFI** | **TLI** | **RMSEA** | **SRMR** | **α** |
| Attention to Body Shape Scale (ABS)^†^ | Attention to body shape | 0.99 | 0.98 | 0.09 | 0.04 | 0.86* | 0.99 | 0.98 | 0.07 | 0.03 | 0.85* |
| Social Physique Anxiety Scale (SPAS) | Comfort about body presentation  Expectation of negative physical evaluation | 0.98 | 0.97 | 0.07 | 0.04 | 0.74-0.88* | 0.96 | 0.95 | 0.09 | 0.05 | 0.77.086* |
| Body Checking and Avoidance Questionnaire (BCAQ)^‡^ | Body checking and avoidance | 0.96 | 0.95 | 0.08 | 0.08 | 0.84-0.99* | 0.98 | 0.98 | 0.08 | 0.07 | 0.79-0.97* |
| Body Satisfaction Scale (BSS) | Satisfaction with body and muscles^¶^  Dissatisfaction with body and fat^║^  Satisfaction with external body parts | 0.98 | 0.97 | 0.09 | 0.06 | 0.82-0.90* | 0.99 | 0.99 | 0.05 | 0.03 | 0.74-0.91* |
| Psychosocial Impact of Dental Aesthetics Questionnaire (PIDAQ) | Dental self-confidence  Social impact  Psychological impact  Esthetic concern | 0.95 | 0.94 | 0.08 | 0.07 | 0.70-0.90* | 0.96 | 0.95 | 0.08 | 0.06 | 0.72-0.90* |
| Orofacial Esthetics Scale (OES)^§^ | Satisfaction with Orofacial Appearance | 0.96 | 0.95 | 0.11 | 0.04 | 0.90^#^ | 0.97 | 0.96 | 0.10 | 0.04 | 0.90^#^ |
| Satisfaction with Life Scale (SWLS) | Life satisfaction | 0.99 | 0.99 | 0.03 | 0.02 | 0.85* | 0.98 | 0.97 | 0.06 | 0.02 | 0.86* |

CFI: Comparative Fit Index, TLI: Tucker-Lewis Index, RMSEA: Root Mean Square Error of Approximation, SRMR: Standardized Root Mean Square Residual. *α: ordinal alpha coefficient. ^#^α: Cronbach’s alpha coefficient. ^†^Refined model without item 3. ^‡^Refined model with correlation between errors of items 5 and 8. ^§^Refined model with correlation between errors of items 1 and 2. ^¶^Dimension measured only in the male subsample. ^║^Dimension measured only in the female subsample.
