# Supplementary figures and images for: Impact of body and orofacial appearance on life satisfaction among Brazilian adults
Source: PLoS One. 2022 Nov 4;17(11):e0275728. doi: 10.1371/journal.pone.0275728 (PMC9635715; doi:10.1371/journal.pone.0275728)

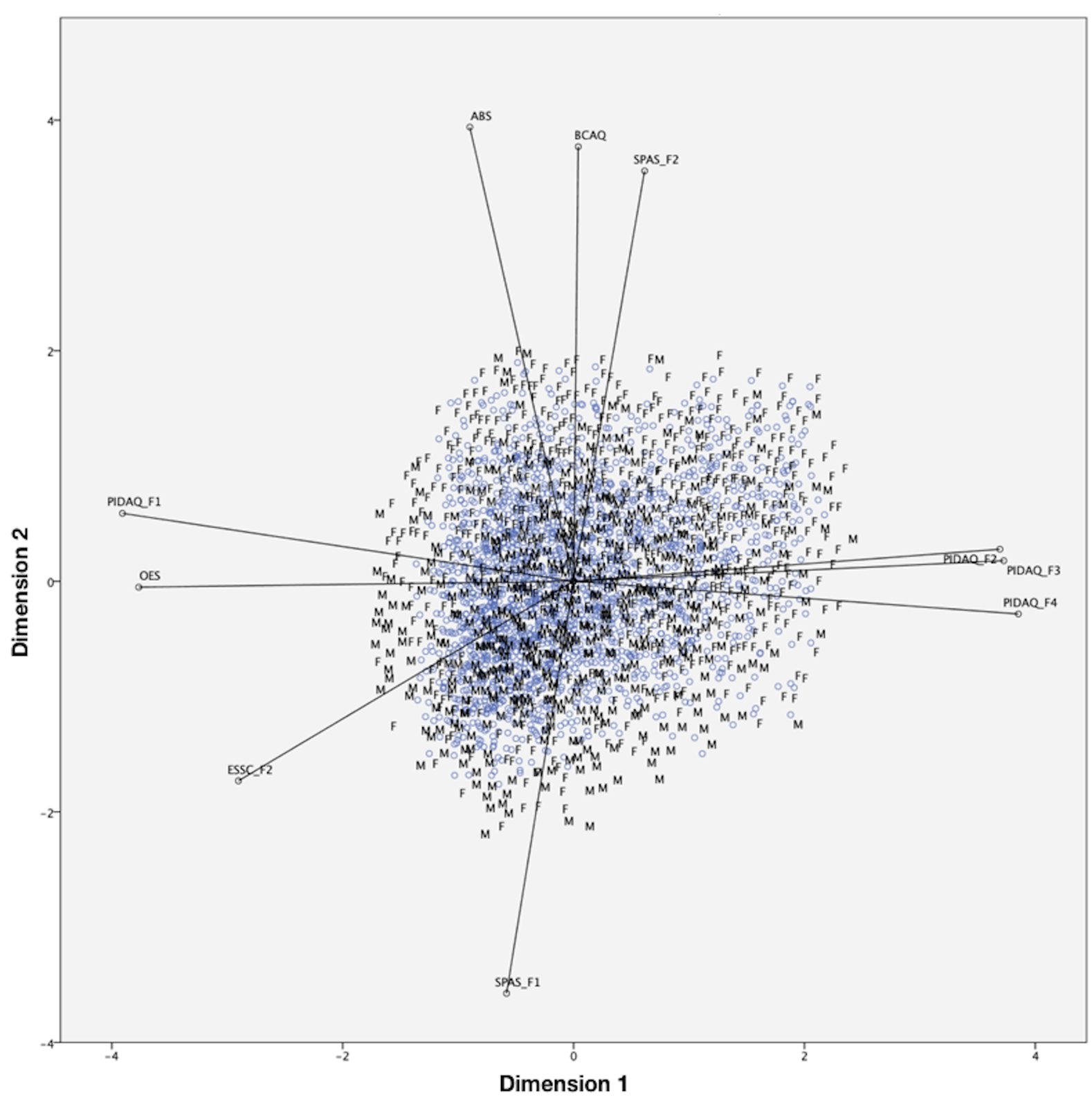

Supplement: S1 Fig — M: male; F: female; ABS: attention to body shape measured by Attention to Body Shape scale; SPAS_F1: comfort about body presentation measured by the Social Physique Anxiety Scale (SPAS); SPAS_F2: expectation of negative physical evaluation measured by SPAS; BCAQ: body checking and avoidance assessed by the Body Checking and Avoidance Questionnaire; ESSC_F2: satisfaction with external body parts measured by the Body Satisfaction Scale; PIDAQ_F1: dental self-confidence measured by the Psychosocial Impact of Dental Aesthetics Questionnaire (PIDAQ); PIDAQ_F2: social impact measured by PIDAQ; PIDAQ_F3: psychological impact measured by PIDAQ; PIDAQ_F4: esthetic concern measured by PIDAQ; OES: satisfaction with orofacial appearance measured by the Orofacial Esthetics Scale. (TIF) [file pone.0275728.s002.tif]
