# Supplementary material for: Impact of body and orofacial appearance on life satisfaction among Brazilian adults
Source: PLoS One. 2022 Nov 4;17(11):e0275728. doi: 10.1371/journal.pone.0275728 (PMC9635715; doi:10.1371/journal.pone.0275728)
Supplement: Attachment — Submitted filename: Response to Reviewers.docx [file pone.0275728.s004.docx]

PONE-D-22-14345

Impact of body and orofacial appearance on life satisfaction among Brazilian adults

Dear Dr. Manoelito Ferreira Silva Junior,

Academic Editor PLOS ONE

Thank you for reviewing our manuscript and considering the study for publication after the requested review. We are submitting a revised manuscript highlighting the changes and a clean version. Please kindly see below our response point-by-point to reviewers’ comments and suggestions.

We hope that the responses and the revised manuscript address the reviewers' concerns.

Sincerely,

The Authors

| Reviewers' comments:  Reviewer's Responses to Questions | Authors’ Responses |
| --- | --- |
| 1. Is the manuscript technically sound, and do the data support the conclusions?  The manuscript must describe a technically sound piece of scientific research with data that supports the conclusions. Experiments must have been conducted rigorously, with appropriate controls, replication, and sample sizes. The conclusions must be drawn appropriately based on the data presented.  Reviewer #1: Yes  Reviewer #2: Yes | Thank you. |
| 2. Has the statistical analysis been performed appropriately and rigorously?  Reviewer #1: Yes  Reviewer #2: Yes | Thank you. |
| 3. Have the authors made all data underlying the findings in their manuscript fully available?  The PLOS Data policy requires authors to make all data underlying the findings described in their manuscript fully available without restriction, with rare exception (please refer to the Data Availability Statement in the manuscript PDF file). The data should be provided as part of the manuscript or its supporting information, or deposited to a public repository. For example, in addition to summary statistics, the data points behind means, medians and variance measures should be available. If there are restrictions on publicly sharing data—e.g. participant privacy or use of data from a third party—those must be specified.  Reviewer #1: Yes  Reviewer #2: Yes |  |
| 4. Is the manuscript presented in an intelligible fashion and written in standard English?  PLOS ONE does not copyedit accepted manuscripts, so the language in submitted articles must be clear, correct, and unambiguous. Any typographical or grammatical errors should be corrected at revision, so please note any specific errors here.  Reviewer #1: No  Reviewer #2: Yes | The language has been revised by a professional company (Semantix, project number 76018646). |
| 5. Review Comments to the Author  Please use the space provided to explain your answers to the questions above. You may also include additional comments for the author, including concerns about dual publication, research ethics, or publication ethics. (Please upload your review as an attachment if it exceeds 20,000 characters) |  |
| Reviewer #1  Abstract |  |
| Authors should it clear which form of evaluation, if it occurred through questionnaires. | We added this information in the Abstract (methods). |
| In the results, the authors should add the real value of p in the sentence: “For men, the affective and satisfaction components of BI had a greater impact on life satisfaction than the other factors. For women, both BI and OA had a similar impact.” | We added to the abstract information regarding the impact value (β) and p-value. |
| Introduction  The introduction is too long.  “…studies aiming to evaluate different body image components...” Missing the studies reference.  “Measurement of the body image is challenging, as it requires instruments capable of capturing body valuation from the individual's perception and/or attitudes.” Missing the reference.  “However, studies that evaluate body image usually do not include orofacial …” Which studies?  “OES assesses satisfaction with orofacial appearance and PIDAQ assesses the impact that dental esthetics have on the individual's life, such as self-confidence, social impact, psychological impact, and concern about the appearance of teeth.” Missing the reference. | We understand that the introduction may be a bit long, however, our manuscript involves concepts that cross areas of knowledge related to body image, dentistry, psychology, and psychometrics. Since Plos One has a broad readership from many different fields of knowledge, we believe it is important to point out some main concepts that may not be usual for all readers.  We added the references in the sentences that the reviewer pointed out. |
| Methods  The information about the measuring scales and sample characterization would be clearer if they were present in tables. | Thank you for the suggestion. Regarding the sample characterization, Table 1 presented in the Results already contained most of the variables. To avoid overlapping information by adding a new table with this same information, we just included all the characterization variables (and their results) in Table 1 and cited this table in Methods - Sample Characterization.  Regarding the measured scales, we have removed the psychometric indicators of each scale for each sample from the text (Methods - Scale Measurements). These data have now been presented as supporting information (S1 Table), which also includes the dimension measured by each instrument. |
| Discussion  An English grammar review is required throughout the manuscript. | The language of the manuscript has been revised by Semantix (project number 76018646). |
| Conclusion  At the beginning of the conclusion, the authors describe the results. Authors should better structure the conclusion without repeating the results found by the study. | The conclusion was restructured. |
| Reviewer #2: Dear authors, thank you for the submission. The manuscript is well-written and well-structured, however, there are some points that must be improved, described below. | Thank you. We changed the issues pointed out by the reviewers to improve the manuscript and make it suitable for publication. We hope we have met them. |
| L.69-70: “These behaviors can be enhancers for the onset of mental disorders such as eating disorders [2]” What other disorders are related to the behaviors mentioned? Contextualize with what was reported. | The sentence was rewritten adding other mental disorders and contextualizing the relationship. |
| L.112-114: “no studies have been published that evaluate this relationship considering both body and facial appearance simultaneously” This is a poor justification of your study. I recommend improving the justification with precise arguments. It seems to me that the narrative described in the previous paragraph is configured as an adequate justification. | We rewrite the sentence to describe a justification using the argument presented in the previous paragraph. |
| The methods performed are adequate and realized according to validated questionnaires. However, it was mentioned that one item of ABS assess was excluded and not considered for mean score calculation. Could this exclusion be considered some bias for calculating the mean, and consequently affect the result? | Thank you.  We understand the reviewer's concern. But it is actually the maintenance of this item that could be considered a bias in obtaining a score for attention to body shape (measured by ABS). This is because, after conducting Confirmatory Factor Analysis on the study sample data (results in S1 Table), this item presented low factor loading. In other words, this item presents a possible local fit problem, suggesting that the measured construct (attention to body shape) is not reflected in this item for the study sample. We have therefore decided to exclude this item in order to ensure that the estimates obtained are valid and reliable.  In the manuscript (Methods - Measurement Scales - 1st paragraph), we added a sentence justifying that the exclusion of the item was to avoid bias and ensure data validity and reliability. |
| L.509-10: “Individuals with higher BMI values had higher levels of the negative components of body image and lower levels of the positive components” Please, insert the classification of BMI in the Methods section. | Based on previous studies that evaluated the impact of BMI on well-being (*Swami et al. Positive body image is positively associated with hedonic (emotional) and eudaimonic (psychological and social) well-being in British adults. J Soc Psychol. 2018;158(5):541-52. Frederick et al. Correlates of appearance and weight satisfaction in a U.S. National Sample: Personality, attachment style, television viewing, self-esteem, and life satisfaction. Body Image. 2016;17:191-203. Davis et al. The role of body image in the prediction of life satisfaction and flourishing in men and women. J Happiness Stud. 2020;21(2):505-24.)*, we clarify that in the present study we chose to use BMI quantitatively (Kg/m^2^) and not considering categories obtained by classification.  We added a sentence at the end of the last paragraph of the Methods-Sample Characterization to clarify this information. |
| L.526-28: “Thus, we expect that the results of this study could help health care professionals to develop and improve a holistic view of human subjects, which would assist the development of patient-centered treatment plans” This is an important aspect that is absent in the discussion topic. Consider inserting the impact of your findings on the population’s quality of life and the inclusion of public policies or the role of health care for these individuals. | Thank you for the suggestion. We added a paragraph discussing what the reviewer pointed out. |
